# Enhancing volleyball training: empowering athletes and coaches through advanced sensing and analysis

**DOI:** 10.3389/fspor.2024.1326807

**Published:** 2024-04-16

**Authors:** Fahim A. Salim, Dees B. W. Postma, Fasih Haider, Saturnino Luz, Bert-Jan F. van Beijnum, Dennis Reidsma

**Affiliations:** ^1^ Digitalization Group, Irish Manufacturing Research, Mullingar, Ireland; ^2^ Human Media Interaction, University of Twente, Enschede, Netherlands; ^3^ School of Engineering, The University of Edinburgh, Edinburgh, United Kingdom; ^4^ Usher Institute, The University of Edinburgh, Edinburgh, United Kingdom; ^5^ Biomedical Signals and Systems, University of Twente, Enschede, Netherlands

**Keywords:** smart sports, digital sports technologies, sports telematics, telematics applications, training methods, action recognition

## Abstract

Modern sensing technologies and data analysis methods usher in a new era for sports training and practice. Hidden insights can be uncovered and interactive training environments can be created by means of data analysis. We present a system to support volleyball training which makes use of Inertial Measurement Units, a pressure sensitive display floor, and machine learning techniques to automatically detect relevant behaviours and provides the user with the appropriate information. While working with trainers and amateur athletes, we also explore potential applications that are driven by automatic action recognition, that contribute various requirements to the platform. The first application is an automatic video-tagging protocol that marks key events (captured on video) based on the automatic recognition of volleyball-specific actions with an unweighted average recall of 78.71% in the 10-fold cross-validation setting with convolution neural network and 73.84% in leave-one-subject-out cross-validation setting with active data representation method using wearable sensors, as an exemplification of how dashboard and retrieval systems would work with the platform. In the context of action recognition, we have evaluated statistical functions and their transformation using active data representation besides raw signal of IMUs sensor. The second application is the *“bump-set-spike” trainer*, which uses automatic action recognition to provide real-time feedback about performance to steer player behaviour in volleyball, as an example of rich learning environments enabled by live action detection. In addition to describing these applications, we detail the system components and architecture and discuss the implications that our system might have for sports in general and for volleyball in particular.

## 1 Introduction

The integration of Sensing and Interaction technology has become increasingly pivotal in sports training and education, catering to athletes across all levels, from amateurs to Olympians. Coaches, trainers, and athletes alike rely on technological advancements to meticulously track and enhance their performance (1–4). This synergy between sports data and interaction technology yields a plethora of applications, offering invaluable insights for refining training methodologies [e.g., (5–7)]. Furthermore, it lays the groundwork for innovative interactive sports exercises [e.g., (8–10)].

Interactive systems such as Dashboard-style retrieval systems stand out as a prevalent design choice, facilitating streamlined access to video summaries via database queries (11). These systems aim to enrich videos with supplementary information, ranging from overviews and visual annotations to data-driven tables and graphs, thereby enabling deeper insights (12–14). The underlying database may house manually tagged events or those detected through (semi-)automated means (3, 15–18), harnessing the potential of data-driven insights to inform decision-making processes. While traditionally aiding coaches and trainers, these systems also offer automation possibilities (10, 19, 20), facilitating post-training and game analysis interactions.

Human pose estimation and performance analysis plays a crucial role in technology enhanced sports training. In recent years, researchers have made significant strides in human pose estimation, tracking, and recognition. Notably, Puwein et al. (21) proposed a method that jointly estimates camera and human poses from multi-camera videos with wide baselines. Zhao et al. (22) introduced the Semi-Supervised Discriminant Analysis with Global Constraint (SDG) algorithm, enhancing pose estimation accuracy using both labeled and unlabeled data. Jalal et al. (23) focused on 3D pose estimation from RGB-D video sequences, leveraging a generative structured framework. Additionally, SVMs (24) play a crucial role in pose recognition. Complex human activity recognition (25, 26) and Kinect-based systems (27) and visual word extraction (28) further contribute to this evolving field. These insights inform practical applications and inspire ongoing research efforts.

When used in real time during the training activity, real time visualisation of the athlete’s performance can be used to steer their execution of an exercise, by representing the summarized measurements directly, or in a comparison with ideal schedules or past self or peer performance [e.g., (29, 30)].

Sports interaction technology can also be used in real time during training to adapt the training session, that is, to modify the training or steer the player’s behavior to enhance the instant training experience. These interactive systems use specialized hardware [e.g., (31)] where the moving body and the data derived from it form the interface through which athletes interact with digital-physical exercise systems, by providing the input triggers to which the system should respond in interaction. This leads to rich learning environments that allow for better motor learning.

All of these types of applications of sports data and interaction technologies, ranging from post-hoc analysis to online, real-time interaction, build upon an underlying layer of sensor data processing. Furthermore, each of these types of application sets its own requirements for the sensor data processing in terms of speed and accuracy as well as in terms of the type of recognition errors that may be considered acceptable or problematic.

Our aim is to provide an integrated platform that combines the various requirements to offer access to data analytics and longitudinal modelling for post-hoc analysis as well as functionalities for real-time online sensor based interaction to create new forms of sports training.

The work presented in this paper is one of the outcomes of the Smart Sports Exercises (SSE) project. The project aimed to extend the state of the art by combining sensor data, machine learning and pressure sensitive in-floor displays to create new forms of volleyball training and analysis. The sensors are used to model the behaviour of volleyball players for analysis and to design interventions to provide tailored feedback (32, 33). User experience should be enhanced in these systems through instant multimodal and interactive feedback (2, 34–36). To this end, the project utilizes a layered approach to first detect and classify individual volleyball player actions from raw sensor data. The detected and classified actions serve as input for the next layer which recognises multiplayer actions such as volleyball rallies or the so called “volleyball complexes” [cf. (37)] in order to model (group) behaviour across volleyball matches and training sessions (38) and to generate relevant feedback for players and coaches.

The current paper presents an extension of our previous work (3) in which we presented a system that automatically classifies volleyball actions performed by players during their regular training sessions, supplementing video recordings to allow coaches and players to easily search for the information or event of interest (e.g., all the serves by a particular player). While providing the results in real time, the earlier system was designed for sports data analysis and feedback and not for technology enhanced sports training. Here we go beyond that earlier work and describe the design and components of an end-to-end pipeline which can use different module configurations to create a sensor driven, interactive, volleyball training environment while also allowing us to collect data to allow efficient post-hoc exploration of events of interest for coaches and trainers and other participants.

The contributions of this paper include the following:

• a description of a modular and easy-to-use sensing and interaction technology pipeline for volleyball training;
• the showcasing of said pipeline through the development of a Bump, Set, Spike exercise scenario to enhance volleyball training with wearable sensors and experimental in-floor display hardware;
• experimentation with deep learning techniques to detect and classify volleyball actions instantly (online) from within the proposed platform, and
• a discussion of implications of our results for sensor data processing in context of sports interaction technology.

The rest the paper is organized as follows. Section 2 describes the related work. The architecture of the modular system is described in Section 3. The system is implemented and experimentation is performed using the Bump, Set, Spike game described in Section 4. The experimentation with deep learning and results are described Section 6. Section 6.5 discusses the results and its applications while Section 7. ends with conclusive remarks and future directions.

## 2 Related work

Sports data technology can roughly be characterised along the data science lifecyle as applied to sports data:

$$\text{Collect}\to \text{Maintain}\to \text{Clean}\&\text{Process}\to \text{Analyze}\&\text{Model}\to \text{Communicate}\&{\text{Use}}^{1}$$

Measurements of[Fn FN0001] sports behaviours and activities are part of the first few steps, involving a wide range of platforms such as smart watches, Inertial Measurement Units (IMU), camera based tracking systems, Local Positioning Systems, manually obtained game statistics and player self reports, and various other body worn and environmental sensors. The middle steps comprise, among other things, storage of measured and processed data in a transparent and interoperable way and the various ways to recognize and mathematically model the data, including modeling patterns in individual athletes behaviours and team patterns of behaviours. The last step concerns the interaction, disclosing the data, and retrieval and sense making of the content, to support athlete and coach in better understanding their performance and making decisions to improve training regimes and competition strategies.

In this section, we first delve into the utilization of various sensors, with a particular emphasis on IMUs, for detecting events of interest in sports activities. Subsequently, we shift our attention to the communication and utilization stage. Here, we explore dashboards, retrieval systems, and sports interaction technology, all of which play crucial roles in enhancing play and learning experiences. Lastly, we delve into the context within which these activities occur—namely, sports learning and performance. This topic places significant emphasis on the aspects we must measure and model to enhance athletic outcomes.

### 2.1 Automatic detection of events of interest in sports as a necessary foundation

All applications for the use of sports data discussed in this paper depend at least partially on automated analysis of the sports content.

Many published papers are concerned with ways to measure and model individual behaviour (among others, for position tracking and action recognition, performance and fatigue detection, and action *quality* recognition) e.g., a recent survey on human movement quality assessment (40). These papers focus on the measurement and tracking devices that can be used in various sports [e.g., (41, 42)], including highly customized measurement systems, for example for climbing holds (43) or bowling grips (44). Other papers focus on what is detected or analysed with the resulting measurements. This is done through many different methods and sensors, camera based, environment embedded sensors, or body worn sensors (45). Especially wearable IMUs [e.g., (46)] are becoming increasingly popular for sports related action analysis because of their reasonable price as well as portability (47). While researchers have proposed different configurations in terms of number and placement of sensors (48), it is ideal to keep the number of sensors to minimum due to issues related to cost, setup effort and player comfort (48–51).

In recent years, Human Activity Recognition (HAR) has gained prominence across various domains, including medicine, education, entertainment, and visual surveillance. Researchers have explored both traditional machine learning techniques and modern deep learning architectures for classifying human activities. Notable studies include (52) comprehensive survey, which discussed dataset characteristics and action recognition methods. (53) proposed a deep learning-based framework using Harris corner points and histograms for interaction recognition. Kamal et al. (54) focused on 3D human body detection and tracking, utilizing spatiotemporal features and a modified Hidden Markov Model (M-HMM). The supervised framework proposed by Chattopadhyay and Das (55) excelled in recognizing interactions between performers. Lastly (56, 57) emphasized the importance of labeled body parts in depth silhouettes for accurate 3D activity detection using self-organized maps. These contributions collectively advance our understanding of HAR and pave the way for future research.

For sports training, IMUs are a popular type of measurement device, being used for, among other purposes: detecting skiing jumps (58), turns (59) and general movements (15); snowboard aerial acrobatics (60); volleyball serve type (61), spike skill (62) or general action type (3, 34); horse riding quality through hip asymmetry (63); discriminating several common domestic activities from several common sports activities (1); calculating soccer shot/pass statistics (64); detecting swimming breast stroke phases (65); training session activities and possible injuries (66); (table) tennis strokes and actions (18, 47, 67, 68) and serve quality (69); lunge and deadlift biomechanics (70, 71); skateboard tricks (72); rugby activities (73); field hockey activities (74); movement in arm swings in golf (75) and baseball (76); and basketball movements, activities, and poses (77, 78).

Other types of sensing devices are also used to detect events in sports. For instance, Chi et al. (79) employs piezos to detect scoring hits in taekwondo. Video technology has proven valuable across a wide range of tasks, from segmenting and classifying tennis strokes (80) to identifying skateboarding tricks (81). Additionally, the ubiquity of smartphones enables their use in identifying sports-related activities in soccer, hockey (82), and basketball (83). Moreover, scholars like (84) leverage the Catapult system to delve deeply into the precise capture of athletes’ movements in team sports, employing Local Positioning Systems (LPS). In a similar vein, Kesicki and Lewicki (85) utilize GPS tracking to assess the physical fitness of football players.

Other works are about measuring and modelling *group* behaviour in sports. For example, Loureiro et al. (86), Drikos (87), Hurst et al. (88) and Laporta et al. (89) modelled patterns of group interaction in volleyball teams in various ways based on manual annotation; Bagautdinov et al. (8) use a neural network approach to detect individual activity to infer joint team activities in the context of volleyball games; Beenhakker et al. (38) built on that to show how such models can be made useful for Sports ITech purposes but also showed how the usefulness depends very much on the quality of the underlying action recognition; and Lamas et al. (90) created a computational system to model and simulate team strategies as patterns of individual athlete actions.

Various detection and modeling solutions employ diverse approaches, differing in their online or offline nature and real-time capabilities, as well as computational requirements. Moreover, specific parameterizations of a method can influence the algorithm’s typical errors. For instance, one may prioritize minimizing false negatives over false positives, tolerate occasional single errors as long as overall statistical accuracy remains intact, or concentrate on distinguishing between specific subsets of categories more than others. These choices largely hinge on the intended application of the detection algorithms. The subsequent sections delineate two primary application categories of sports data.

### 2.2 Dashboards and sports data retrieval systems

Dashboards and retrieval systems offer the athlete or coach quick access to sports data — and through the data often also to salient recordings of past training situations — to base training programs, match strategies, and other decisions on, often to optimize the athlete’s performance. Stein et al., who published extensively on visual sports data analytics, discuss how this step is about *making sense of the data*, from analysing the data to re-representing it and disclosing it in a way that contributes real insight. This is not only about ways to find out *what* situations and events happened, but very importantly also about gaining insight in *when* and *why* these happened (91).

Dashboard and retrieval systems may focus on the (elite) sports context or may be embedded in the context of sports pedagogical processes (92, 93). For example, a video recording analysis can show different events of interest which may help to get insightful tactical play and player engagement (92). Video-edited game analysis is a common method for post-game performance evaluation (93). Accessing events of interest in sports recordings is of particular interest for sports fans [e.g., a baseball fan wishing to watch all home runs hit by their favourite player during the 2013 baseball season (17)] as well as for coaches [e.g., a coach searching for video recordings related to the intended learning focus for a player or the whole training session (93)]. Koekoek et al. developed an application named Video Catch to manually tag sports actions during matches and training sessions (93), which can then be used as input to discussions of strategy, learning plans, or technique. However, these examples require events to be manually tagged which not only requires time and effort but also distracts a trainer’s attention from training to tagging. Other systems therefore aim to automatically tag events to avoid manual effort and allow trainers to keep a singular focus.

The basic setup of dashboard and retrieval systems is often that they offer access to video summaries of sports activities based on queries to a database which has been filled through manual and automated analysis of what happened in a match or training. Papers may focus on the architectural considerations of sports data retrieval and dashboard systems, such as requirements related to the real-timeness, high data throughput, and distributed nature of having multiple data-sourcing processes; architectural aspects of the processing pipeline, and GUI design considerations [e.g., (15, 17, 18)], or on the indexing and retrieval itself. A somewhat older, but still relevant survey of trends in video retrieval in sports is given by Kokaram et al. (11).

Other work focuses on the design and evaluation of the sensemaking that a coach or analyst carries out with the help of this database of video and data, to turn data into insights and decisions – building on automated but also manual analysis and annotation of sports data. The extensive availability of commercial systems for manual annotation of sports data recordings that are coupled to video retrieval interfaces confirms from sports practice the need for access to objective match data via dashboards and video retrieval interfaces. Sensemaking systems for sports data often support the capture of video and other data, allow for coding the data (manually and automatically), augment the video with visual information to make the video more informative at a glance, offer access to the data to derive new insights and allow users to annotate and enrich the data and video for more insight. To enrich the video recordings, systems allow the analyst to draw over the video (trajectories, highlights, etc); to overlay the videos of multiple occurrences of the same action or show time aligned side by side videos of different moments to compare them visually; visualise the formation, the spatial relation between positions of players in movement through lines, distances, planes and angles; gathering all instances of a certain type of event in one overview; etc. To this end also novel visualisation are researched to give insight-at-a-glance for further exploration. See Perin et al. (12) for a survey; for example Polk et al. (13) describe in detail how three novel visualisation forms allowed two coaches to quickly formulate novel insights regarding their own players; Correia et al. (94) describe how VR can enrich the video material further.

Interfaces for easier access to the data do not only revolve around novel forms of visualisation but also around novel forms of *querying* the large datasets. For example, Shao et al. (14) describes a sketch-based retrieval of field situations in soccer. This allows the query input side to go “plain beyond database queries”, which allows an analyst to ask fairly complex questions in a novel and intuitive way (in their case: asking for a type of spatial situation, rather than a list of recognized events). Then, the results need to be communicated, and presented to players and others to transfer the insights yielded. Most commercial systems support generating rich illustrated reports with data, visualisations, and video materials.

The next step in the process is to support the decision-making on top of the generated reports. Many systems are set up to leave this step to the coaches/analysts. For example, Polk et al. (13) discuss how “[our] results indicate that useful insights can quickly be discovered [via novel visualisations] and *ad hoc* hypotheses based on these insights can conveniently be tested through linked match videos [and smart database access]”, and Koekoek et al. describe extensively how their video interface should be incorporated by teachers into the overall sports teaching processes including discussion and reflection on the gathered content (93). The decision-making can also partially be automated, which is especially crucial when the feedback is to be provided during the sports activity rather than afterwards.

SAETA (10) offers decision support for maximizing an athlete’s effort while minimizing fatigue based on machine learning on measurements from the athlete and from the environment. Vales-Alonso et al. (20) and López-Matencio et al. (19) use wireless sensor networks to measure and analyse many aspects of athlete performance; on the basis of that their system gives advice on how to fulfil a goal such as keeping a constant heart rate while running, depending on track conditions, slope, and runner profile and performance. Van Delden et al. (30) present their system for providing real-time feedback to rowers based on sensor measurements.

These systems are all interactive, and clearly contribute much to sports science and practice. However, these are only the dashboard-oriented systems; in the next section we look more broadly at other types of sports interaction technology.

### 2.3 Sports interaction technology for training exercises and match performance

Sensor based interaction technologies for innovative sports practices is on the rise. There are mainly two approaches for measured sensor data to contribute to training. On the one hand, the dashboard and retrieval based approaches discussed before allow coaches and athletes to gain more insight into their performance, and thus make better-informed decisions regarding modifications to their match strategies and exercise regime. On the other hand, there are interactive training systems that offer completely new kinds of digital-physical exercises, for improved learning, performance, or engagement of athletes [e.g., (32, 95–99)].

Regarding the latter, the diversity of possibilities is wide. Instances of digital-physical exercises include Football Lab, an interactive soccer-training system (95). In this system, a player is placed in a small soccer field where the ball is passed to sensor placed re-bounders. During the game the players are rewarded according to their number of passes. Furthermore, Holsti et. al. propose a trampoline training game which combines a real training environment with a virtual world (100). In this scheme, players’ action on a trampoline is tracked with a depth camera and projected to the fantasy world allowing a player to see her/his virtual character on the screen. TacTowers is an interactive training equipment which aims to improve psychomotor abilities of athletes, such as handball players (99). In the game environment, players are trained to read each other intentions, to predict the outcome while reacting accordingly.

Typically speaking, sports interaction technology involves the so-called sense-think-act cycle of HCI (101). The system senses input, such as relevant sports actions or behaviours, and decides upon contextually relevant responses for the activity. It delivers responses through multimodal interfaces such as displays, wearable sensors or other novel smart environments.

Take FootballLab (102) as an example in which a football field is surrounded by four smart goals equipped with sensors to track the hit position of the ball, and with speakers and lights to provide the feedback. This setting is designed to encourage players to practice soccer related exercises through gamification. Sports interaction technology often targets different combinations of performance, engagement and learning.

Focusing on performance; biofeedback systems are prominent examples of such systems. They are designed to provide feedback on performance characteristics which are difficult for coaches to observe without the use of technology e.g., the timing of a volleyball smash or cross-over technique in speed skating (103). They also empower athletes to draw their conclusions by providing them with additional information that augments the feedback they receive from coaches (103–106).

Sports interaction technology can also help in training for sports events, which occur infrequently during normal play but are important in terms of performance. The FootballLab (102) allows players to train “passing” at a rate much higher than what would be possible during normal play; many other examples of interaction technology to enhance performance training exist (107–109).

Measuring the behaviour and performance of individual athletes not only provides valuable insights but also facilitates adaptive personalization. This capability is pivotal for maintaining a balanced gameplay experience among players with varying skill levels. Adaptive balancing strategies, such as implementing handicaps or dynamically adjusting difficulty levels (5, 7, 110, 111), enable people of different ages and skill sets to engage in harmonious play (112). Moreover, extending this approach can enhance team coordination, offering potential applications beyond individual gameplay.

### 2.4 Motor learning

Motor learning refers to the process through which individuals acquire new motor skills or enhance existing ones by practicing and assimilating knowledge. As a result, their movements become more automatic and efficient. In recent times, data retrieval tools, dashboards, and interactive sports installations have gained popularity due to their ability to accelerate motor learning and enhance performance. These systems, tailored for specific purposes, target various concepts within motor learning to foster motor competence.

#### 2.4.1 Modelling

Model-based practices are central to motor learning in sports and physical education (113, 114). An archetypal example of a model-based practice is the PE-teacher demonstrating how a certain action should be performed. This type of demonstration is referred to as “expert modelling” or “mastery modelling”. Another form of modelling is “learner modelling” in which the pupils learn by modelling their own behaviour (self-modelling) or modelling the behaviour of a peer (peer modelling) (115). Both approaches uniquely contribute to motor learning. Expert models are an easy way to help learners fathom the nature and form of specific movements, whereas learner models promote self-efficacy among learners (115, 116).

To support model-based practices, various (interactive) technologies have been developed. A popular approach is to use augmented or interactive mirrors to accommodate modelling (117–120). Interactive mirrors are perfectly suited to highlight errors in movement and show what the ideal posture, form or movement execution should look like. Besides this, a unique advantage of interactive mirrors over traditional mirrors is that interactive mirrors allow learners to review their performance *after* movement execution. With advancements in OpenCV (121) and MediaPipe (122), the creation of augmented mirrors is easier than ever before. Augmented mirrors are, however, limited in their application. Augmented mirrors are typically employed in dance (119, 120), aerobics (117), or martial arts (118), but appear to have limited value in more dynamic sports like Basketball, American Football or Volleyball.

Another popular approach to support model-based practices is video capture (93, 97). Coaches and PE-teachers make increasingly more use of (pocketable) cameras to support their training practices; recording video of past performances to accommodate self, peer or expert modelling. Finding and capturing the right moment can, however, be a laborious task - one that redirects the focus of the coach from the learner towards the technology (123). Automatic video tagging - as championed in this paper - might alleviate these issues.

#### 2.4.2 Rich learning environments

While automatic action recognition for automatic video-tagging would have undeniable benefits for model-based learning practices, automatic action recognition could also benefit the creation of *rich learning environments*. Motor learning and skill acquisition are the result of the dynamic interaction between athlete, task and environment (124, 125). Interactive technology can be used to specifically shape the task (99, 102, 126, 127) and the environment (5, 32, 128) in which learning occurs. The better the task and environment are tailored to the characteristics of the learner, the more relevant and rich learning opportunities can be presented. Football Lab (126) is an interactive football installation that allows its players to practice passing. Five minutes of play in Football Lab equals 90 min of game-play in terms of passes. Thus, interactive environments can be shaped to hyper-sample valuable skills. Automatic action recognition taps into that potential. Through automatic action recognition, desirable behaviours can be stimulated and inexpedient behaviour can be dissuaded (32).

## 3 System architecture

We present a novel platform built to gather data during sports activities in such a way that the data can be stored and accessed later, for sports data analytics, but also be analysed on the spot to detect salient events, provide real-time feedback on the athlete’s performance, and shape the interaction between athlete and a rich learning environment for the betterment of motor learning, as discussed in the previous section.

The Smart Sports Exercises system is designed to be modular and extensible. The main architecture is based on a Model View Controller (MVC) pattern (129). The controller module as the name suggests acts as a controller between UI-facing modules i.e., the view and the functional or business logic modules i.e., models. The system is configurable to allow different configurations in terms of sensors and interfaces and other functionalities suitable for the application scenario.

**Figure 1:**
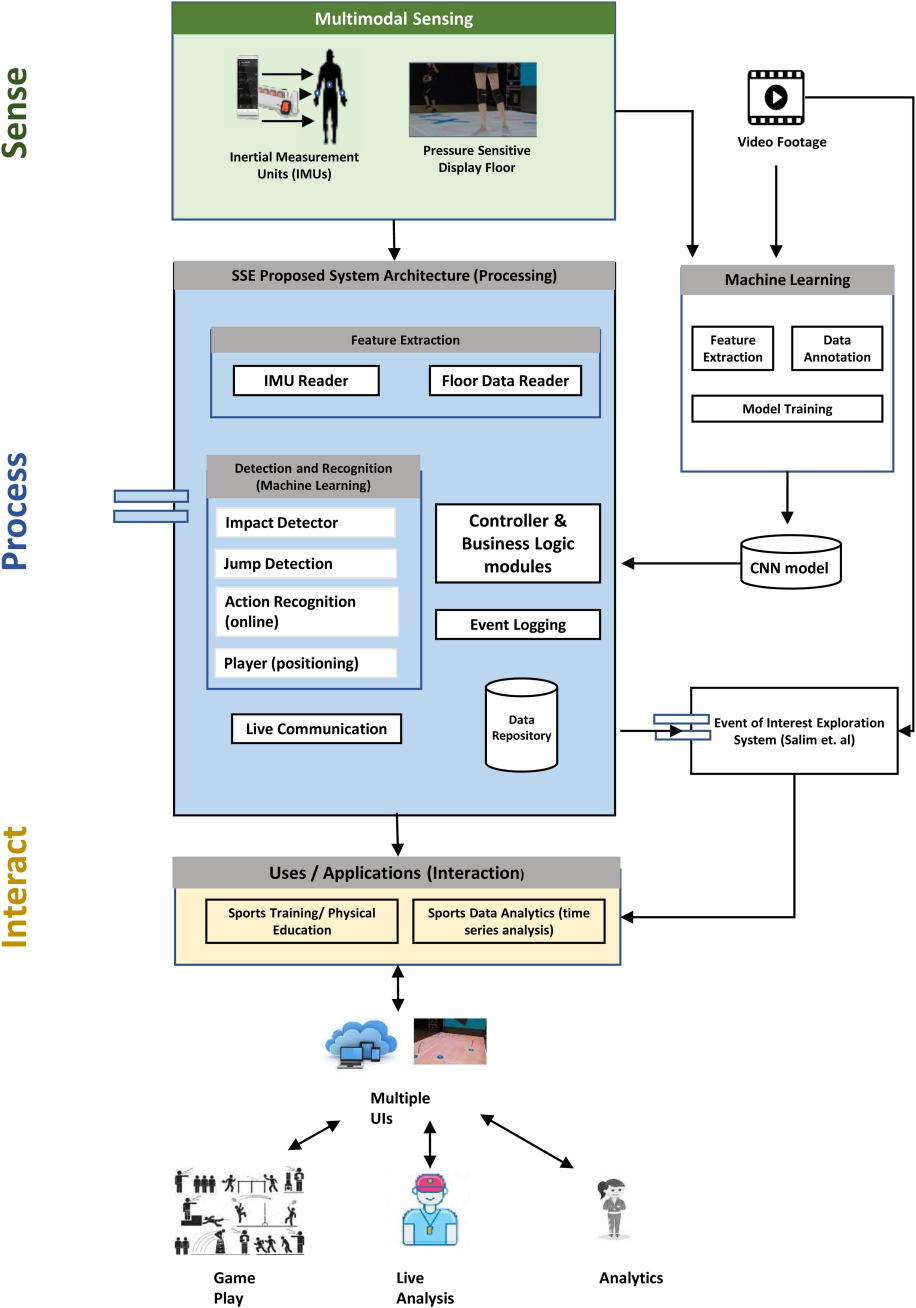
SSE System Block Diagram: During the sensing phase, data is collected from sensors such as Inertial Measurement Units (IMUs) and/or pressure-sensitive floor. In the subsequent processing phase, appropriate modules process this data. Finally, users such as players and coaches can view and interact with the processed information.

Figure 1 shows an overview of the system. Features can be extracted from sensors and processed using online machine learning approaches. Different modules can be configured to allow for suitable interactive sports training and/or store the events of interest in the repository to be available for later analysis and feedback. The machine learning models, finally, can be trained offline using stored recordings of sensor data.

### 3.1 Configuration

The Configuration module determines what modules are to be used in a session and what settings they would have e.g., whether the display floor is being used or not. As the system is designed to be as modular as possible, the configuration module allows the system to be used in different settings with minimum effort in terms of setup. New sensors and their corresponding modules can be added by adding settings to a configuration file that determines what sensors and module configuration to use.

### 3.2 User interface

**Figure 2:**
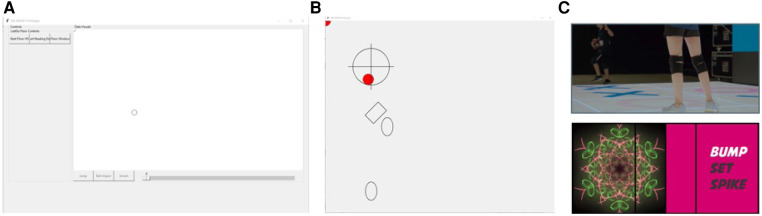
UI screens. (**A**) Control panel on the PC monitor to be used for configuring the display floor UI. (**B**) UI to be displayed on the experimental display floor for player training. The example guides a player on where to step and jump. The red circle shows the player(s) location on the floor. (**C**) UI for the Bump, Set, Spike game to be displayed on the display floor. Progressively complex visuals give cues to the player for the volleyball actions performed.

The user interface components are of two categories. One is a control panel which users can use to control different functionalities. It allows the users to start and stop certain functions e.g., reading data from IMUs and/or floor, whether the user wants to use the Floor display or not, and to display information such as Action Recognition performed etc (see Figure 2A).

The other UI(s) are to be used for player training and game play to be displayed on the experimental pressure sensitive display floor Figure 2B) displays a sample game for volleyball players to train “stepping” and “jumping” by using the markers. The red circle shows the player(s) location on pressure sensitive display floor. Figure 2C displays the sample game “Bump Set Spike” (Section 4) to be displayed on the display floor.

### 3.3 IMU reader

The current IMU reader makes use of “Next Gen IMUs” by X-IO Technologies for detecting the relevant player actions. The sensor reading component reads live data from the Inertial Measurement Units via UDP (User Datagram Protocol). The IMU Reader module reads the stream and filters the data and puts it in queues to be read by the detection and recognition modules. The details of data processing for the IMU signals are described in Section 6.1.

### 3.4 Event logging

Recognized player actions are stored in a data repository. Currently the Solr platform (130) is used for this purpose. Once a player action e.g., “smash” is recognized; it is stored in the solr repository and/or multicasted using UDP. The exact data structure is described previously in Salim et al. (3). The data contains processed information such as event name e.g., “smash” or “jump” and the timestamp of when the event was recognized. The events are used by the events of interest exploration app described in a previous paper (3) and/or by games modules developed by students using the Unity game engine. The Unity game modules read the data by listening to a UDP port.

### 3.5 Action recognition

For most applications of the SSE platform, the action recognition component focuses on the detection of player actions with the ball, based on the data from IMUs that the players wear on their wrists. These events are then communicated and /or stored in the repository based on the configuration. The recognized event such as “smash” is shown on the different UI(s) e.g., A text message is shown on the control panel (Figure 2A) that a “smash” was recognized. The game modules listening on the UDP port upon receiving the event process the information according to their implementation e.g., the sample game displays a pattern on the floor (Figure 2C).

## 4 Implementation of bump set spike game

To illustrate how the architecture works in practice for a specific real-time interactive rich training environment we discuss a showcase training game for practicing Bump-Set-Spike, in which the game mechanics are driven forward by automatically detected volleyball actions from the athletes.

Bump (Forearm Pass), Set (Overhead Pass) and Spike (Smash) are three elementary actions in volleyball – in sequence, these actions form the foundation for an effective attack. Learning to correctly perform these actions is essential for a player at any level and as such it is often the starting point for any volleyball training program. However, especially for younger players, not only is it hard to execute these actions correctly, it is also often not the focus of their play: beginning players from the younger age groups often focus more on “keeping their side clean”, i.e., hitting the ball away over the net as soon as it appears on their side.

One common solution to this, often employed in Dutch youth volleyball training, is to give a team extra score points when they win a rally using a Bump-Set-Spike attack. Although this encourages the proper use of the attack pattern, it is a somewhat heavy-handed approach: the team that is already better at using this pattern –and therefore already has some advantage over the other team– is rewarded with extra points which leads to additional advantage over the other team.

**Figure 3:**
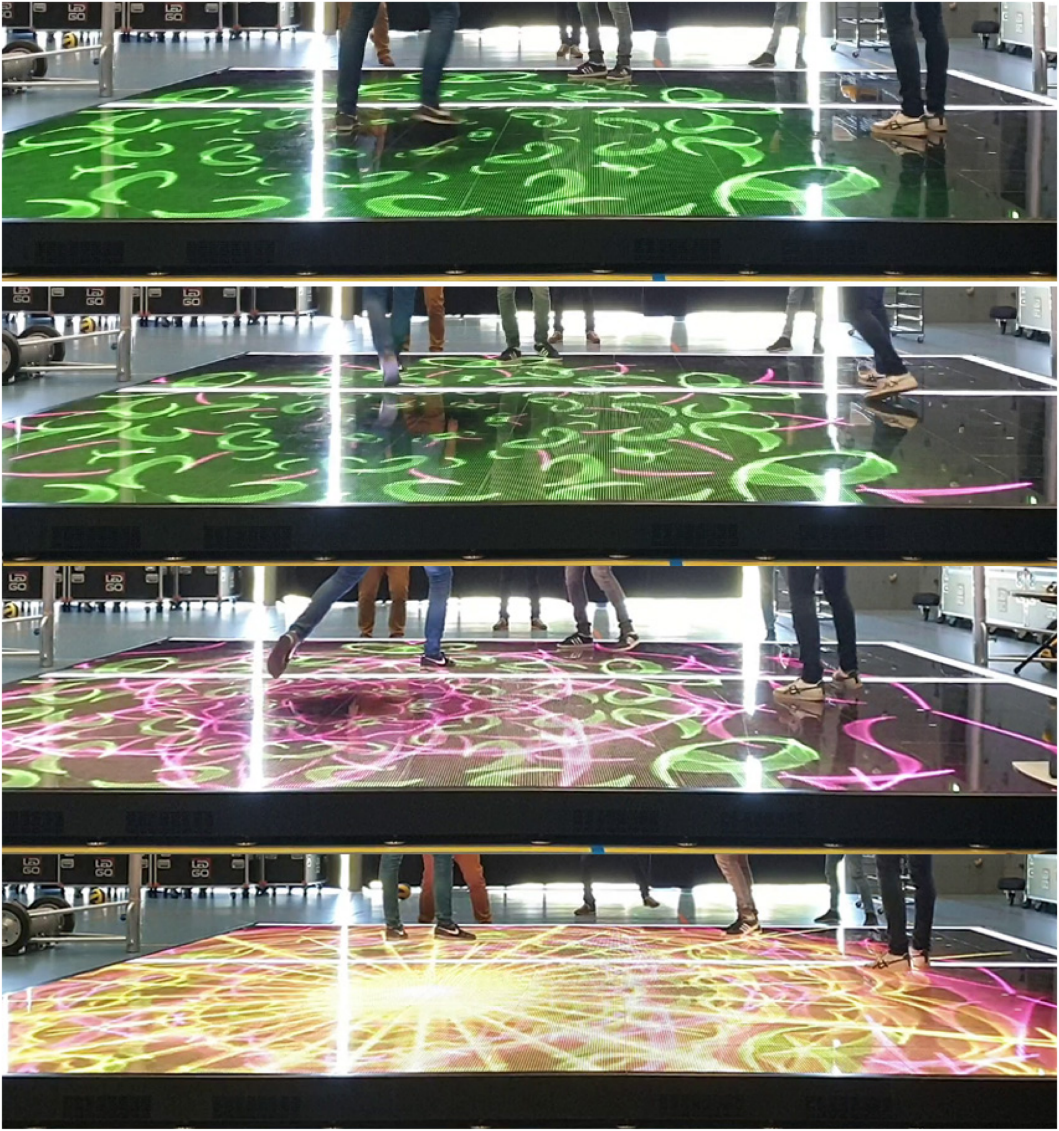
Progressively complex visuals used in the Bump Set Spike Training System.

We propose the Bump-Set-Spike game to address this problem, using a more subtle reward strategy in which proper execution of the attack pattern is rewarded with more beautiful visualisations projected on the floor rather than with any in-game advantage (see Figure 3). This game concept was introduced earlier as one of the conceptual designs in the SSE project (32) and was based on the concept of *enticement* by van Delden et al. (131).

In order to implement this game within the SSE platform, several things are needed. First, a specific combination of sensor modules needs to be configured to gather in-game sensor data and distribute it to the other modules in the system. Second, tailored volleyball action detection modules need to be included, focusing specifically on adequate real-time detection and classification of the bump, set and spike actions. To this end, models have specifically been trained to recognize forearm passes, smashes, and overhead passes from other actions (see Section 6 for more detail). Third, the game interaction module needs to respond to detected actions to turn on or off the “beautiful visualisation rewards”. Finally, in-game detected events as well as the original stream of sensor data can be stored in the database for possible future more detailed game analytics as well as for future further training of the action detection module itself.

## 5 Modifying the system configuration for a different game

Based on the system setup for the Bump-Set-Spike game, only minor modifications are needed to facilitate a different game, for training a better Smash-Jump timing. When jumping for a smash in volleyball, the best timing is to hit the ball at the highest point in the jump, when the player briefly hangs still in the air. It is hard to see for yourself whether your timing is right when you are still learning to do this; to help train this, one could make a game that provides feedback on the relative timing of the jump and the smash and rewards a good execution of the combined action. Here we briefly show how such a training scenario requires only minor adaptations to be able to run it on the SSE platform.

### 5.1 From action classification to simplified smash detection

First, for this game, we need to detect the precise timing of the moment when the hand hits the ball for the smash. The volleyball impact is detected using an IMU worn on the dominant wrist of the player. The approach is similar to Kautz et al. (132). If the z-axis of the accelerometer is below the threshold of -5.1 it is indicative of impact being made by the volleyball. One disadvantage of this approach is that it would also detect an impact if the player claps or high-fives a fellow player. However it did not pose any problem since the deep learning model for action recognition recognized such cases as noise (i.e., non volleyball events).

### 5.2 Jump (timing) detection

The (timing of the) jump is detected by using the signal worn at the back or around the waist. The jump is detected when the z-axis of the accelerometer gives a reading below the threshold of −4. This gives us the end of the jump i.e., the player has jumped and the feet have landed on the ground. From this point backwards in the signal window, we calculate the time point when the player was at the highest point in his/her jump. This is done using the gyroscope signal. The highest point in the jump is detected when the gyroscope x-axis signal changes sign. e.g., if the gyroscope x-axis signal was positive at the end of the jump it would become negative at the high time point of the jump and vice versa.

### 5.3 Game control

Using the two specialised detection components described above, the game logic can be built that offers feedback and rewards to the player depending on how well they time the smash to coincide with the highest point in the jump. Due to the inter-component communication facilities in the SSE platform, this involves catching the messages from the detectors, and then triggering appropriate animations on a screen or on the interactive LED video floor.

## 6 Action recognition modelling and evaluation

Now that we have explained previously, how game scenarios can be implemented, we go deeper into how good action detection works in those setups. We do this on data that was collected earlier with the same sensors in the same setting. Note that data was gathered in training sessions without interaction and regular practice sessions. Yet, by looking at those results we can say something about the expected performance of those modules in our games, and we can speculate on the implications of using those games in actual training settings.

### 6.1 IMU dataset

The dataset utilized for our modeling and experimental analyses aligns with the one employed in the study by Salim et al. (3). For this dataset, eight female volleyball players wore Inertial Measurement Units (IMUs) on both wrists during their regular training sessions. The primary objective was to capture natural gameplay scenarios, ensuring that the collected data accurately represented real-world training conditions.

However, it’s important to note that the dataset exhibits a significant class imbalance. For instance, in the binary classification task of action vs. non-action recognition, we encounter a substantial disparity: 1,453 s of action data compared to a much larger 24,412 s of non-action data (35). This imbalance poses challenges for training robust machine learning models, as it can lead to biased predictions.

To establish ground truth labels, video recordings were synchronized with the IMU data. These videos serve as a reference for annotating specific volleyball actions. Three students, all participants of the eNTERFACE 2019 workshop,^2^ undertook the annotation task using Elan software (133). Given the distinctiveness of volleyball actions performed by players, inter-annotator agreement was remarkably high—there was no ambiguity regarding the labeled actions.

The quality of annotation was assessed through a majority vote mechanism. If all annotators consistently labeled a particular action, it was accepted as the ground truth. In cases where an annotator might have missed or mislabeled an action, the majority consensus prevailed. This rigorous evaluation process ensures the reliability and accuracy of our annotated dataset, which serves as a valuable resource for advancing action recognition research in volleyball.

Training and Testing of the Convolutional Neural Network (CNN) model was performed on the signals from the IMUs sensor worn by players on their wrists. The sampling rate was 40 Hz. The following signals were used from the IMUs:

• 3D Accelerometer x,y,z
• 3D Gyroscope x,y,z
• 3D Magnetometer x,y,z
• 1D Barometer

The window size used is one second, i.e., 40. In short, the input to the CNN was a window of 40×20.

### 6.2 Statistical functions for classification

We have applied basic statistical functions (mean and standard deviation of features and their first-order derivates over the window of one second (i.e., 40 samples × 20 short-time features) resulting in 80 features to represent the window of one second for classification. In this experiment, no frames/windows are discarded.

(1) $$\begin{array}{rl} & \text{UAR}=\frac{1}{\left|C\right|}\sum_{i=1}^{\left|C\right|}\frac{{\text{TP}}_{i}}{{\text{TP}}_{i}+{\text{FN}}_{i}}\\ & \;\;\text{where}\begin{array}{cc} & \text{UAR represents the Unweighted Average Recall.}\;\\ & |C|\text{ denotes the number of classes.}\;\\ & {\text{TP}}_{i}\text{ represents the number of true positives for class }i.\;\\ & {\text{FN}}_{i}\text{ represents the number of false negatives for class }i. \end{array} \end{array}$$

We applied decision tree (UAR = 57.3% with avg. $F_{1}\;\text{Score}$ of 61.54% in 10-fold cross-validation and UAR = 48.76% with avg.$F_{1}\;\text{Score}=45.98\%$ for leave one subject out cross-validation) and linear discrimination classifiers (UAR = 63.8% and averaged $F_{1}\;\text{Score}=66.6\%$ for 10-fold cross-validation and UAR = 62.21% with $F_{1}\;\text{Score}$ of 59.37% for leave-one subject out cross-validation). UAR (as shown in Equation 1), standing for Unweighted Average Recall, represents the average recall value across all classes in a multiclass classification scenario, reflecting the model’s proficiency in accurately identifying positive examples. Meanwhile, the $F_{1}\;\text{Score}$, also referred to as the $F_{1}\;\text{Score}$, offers a comprehensive evaluation of a model’s performance in binary classification by incorporating both precision and recall, calculated as the harmonic mean of these two metrics.

### 6.3 Active data representation for classification

**Figure 4:**
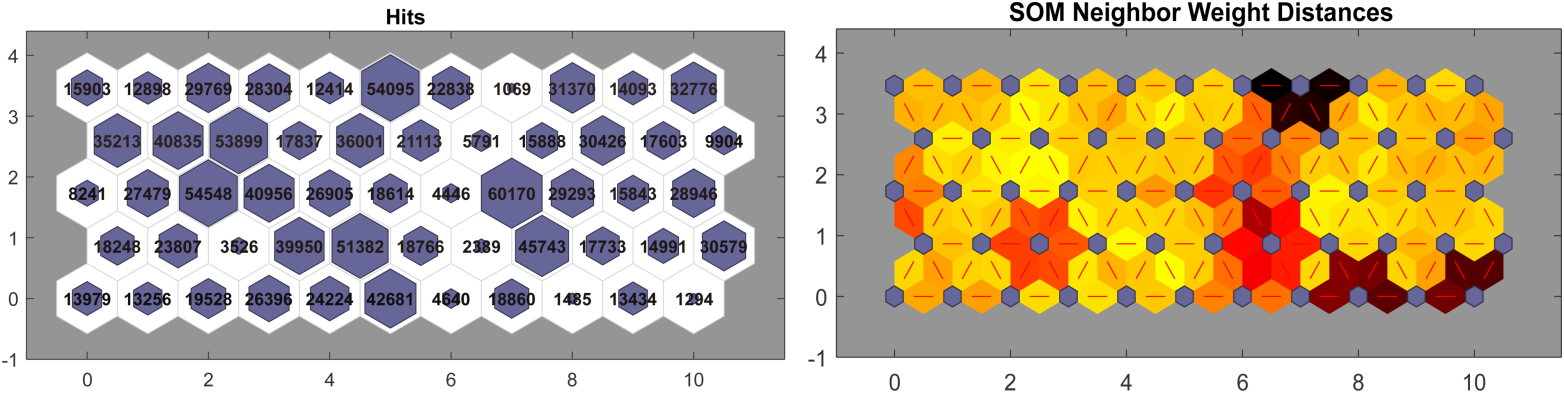
ADR feature extraction model with m=55 which provides the result of 74.47% (UAR) for Action Recognition with LDA classifier. The left figure indicates the number of frames present in each cluster (hexagon i.e., neuron) and the right figure indicates the distance between clusters (blue dots i.e., neurons) and darker colour indicates greater distance between clusters. The red lines connect neighbouring neurons.

**Figure 5:**
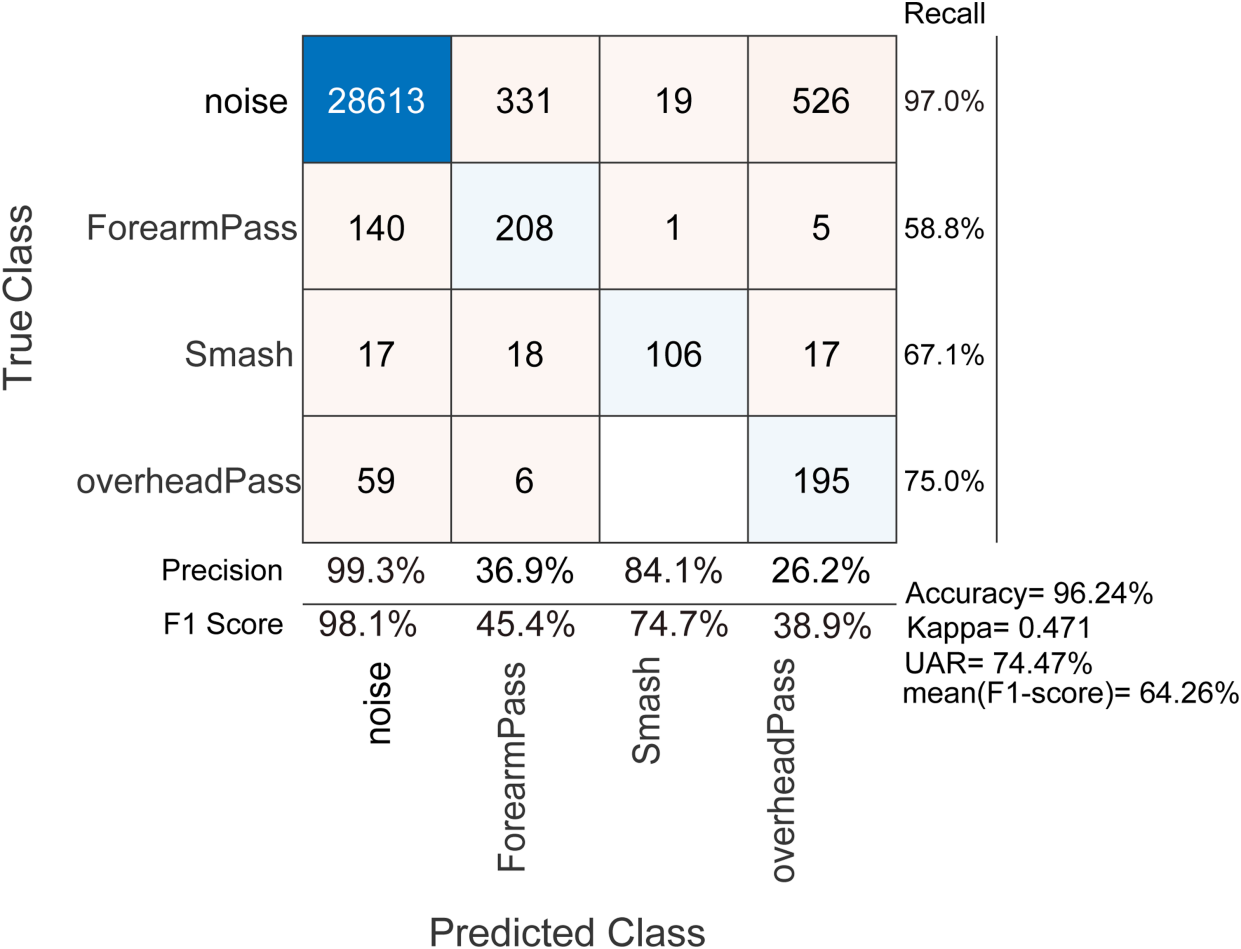
Action Recognition through ADR: 10-fold stratified cross-validation (with random shuffle) Results without impact detection method and no frames/windows discarding.

In this section, we describe our active data representation method briefly (134–136). The ADR method involves the following steps:

1. *Clustering* of frames: Self-Organising Maps (SOM) (137) are employed for clustering of all the frames using 20 short-time features. The number of clusters was determined through a grid search hold-out-validation procedure with a hyperparameter space of m∈{5,10,…,100}. An example of clustering (i.e., feature extraction model) is shown in Figure 4.
2. *Generation* of the Active Data Representation (${\text{ADR}}_{Ai}$) vector is done by first calculating the number of frames in each cluster over 1 s (Ai), that is, creating a histogram of the number of frames ($n{\text{ADR}}_{Ai}$) present in each of the m clusters for every window of one second. Then, to model temporal dynamics we calculate the mean and standard deviation of the rate of change with respect to the clusters associated with the frames for each window ($c{\text{ADR}}_{Ai}$), where the rate of change is given by an approximation of the derivative
  $$v{\text{ADR}}_{Ai}=\frac{\partial c{\text{ADR}}_{Ai}}{\partial t},$$
  with respect to time (t).
  (2) $$n{\text{ADR}}_{Ai_{\mathrm{norm}}}=\frac{n{\text{ADR}}_{Ai}}{\|n{\text{ADR}}_{Ai}{\|}_{1}}$$
3. *Fusion*: the ${\text{ADR}}_{Ai_{\mathrm{norm}}}$ feature set encompasses the features of $n{\text{ADR}}_{Ai_{\mathrm{norm}}}$, and $v{\text{ADR}}_{Ai}$. Therefore, a feature vector with a dimensionality of m+2 is generated to represent each instance (Windowofonesecond) for classification.
4. *Classification*: The classification experiment is performed by linear discriminant analysis (LDA) using ADR features. A 10-fold cross-validation procedure is adopted, and the results are shown in Figure 5. It is found that m=55 provides the best results, which results in a reduction of dimensionality from 800 (20×40) to 57 features. The model for feature extraction is shown in Figure 4.

### 6.4 CNN for classification with impact detection

The input windows (40×20) were created by the following procedure. Once the impact is detected in the signal window using the impact module (see Section 5.1 for details.), the window is created by taking the impact frame as a centre and surrounding frames are taken to create a one-second (40 Hz) window. It could lead to overlapping windows, as there could be multiple frames in a window that are detected as impact frames.

Once the window is taken, the corresponding label is checked from the annotation. If there is an action label found, the window is considered a volleyball action such as a forearm pass, otherwise, it is considered a noise sample. A window with a mix label (noise + action) is discarded.

The signal window is used as input to train the Convolutional Neural Network (CNN) with Adam optimiser and cross-entropy loss. Table 1 shows the trained CNN.

**Table 1:** Convolutional neural network.

Layer type	Output shape	Param #
Conv1D	(None, $n_{\mathrm{timesteps}}-2$, 64)	640
Conv1D	(None, $n_{\mathrm{timesteps}}-4$, 64)	12,352
Dropout	(None, $n_{\mathrm{timesteps}}-4$, 64)	0
MaxPooling1D	(None, $(n_{\mathrm{timesteps}}-4)/2$, 64)	0
Flatten	(None, $(n_{\mathrm{timesteps}}-4)/2\times 64$)	0
Dense	(None, 100)	200,100
Dense	(None, $n_{\mathrm{outputs}}$)	10,100

### 6.5 Results and discussion

The classifiers are evaluated using both 10-Fold stratified cross-validation and Leave One Subject Out (LOSO) methodologies. The 10-Fold cross-validation, used random shuffle where fold contains data from the same subjects. In LOSO cross-validation the folds do not contain data from the same subjects. This setting helps in identifying features/methods that are less invariant to subjects’ characteristics and helps in understanding how method performance is being affected by incorporating personal data for training machine learning.

For 10-fold cross-validation, The ADR method is able to predict the volleyball actions with an accuracy of 96.24%, well over the blind guess of 25% as shown in Figure 5. The proposed architecture also achieved an unweighted average recall (UAR) of 74.47% and an averaged $F_{1}\;\text{Score}$ of 64.26% with a Kappa of 0.471. It is noted that the noise is clearly identified with the highest recall of 97.0%, precision of 99.3% and $F_{1}\;\text{Score}$ of 98.1%. The overhead pass and smash actions achieved a recall of 75.0% and 67.1.1% respectively. We obtained low precision rates for the overhead pass (26.2%) and forearm pass (36.9%), indicating that these actions are often misclassified as noise by the classifiers.

**Figure 6:**
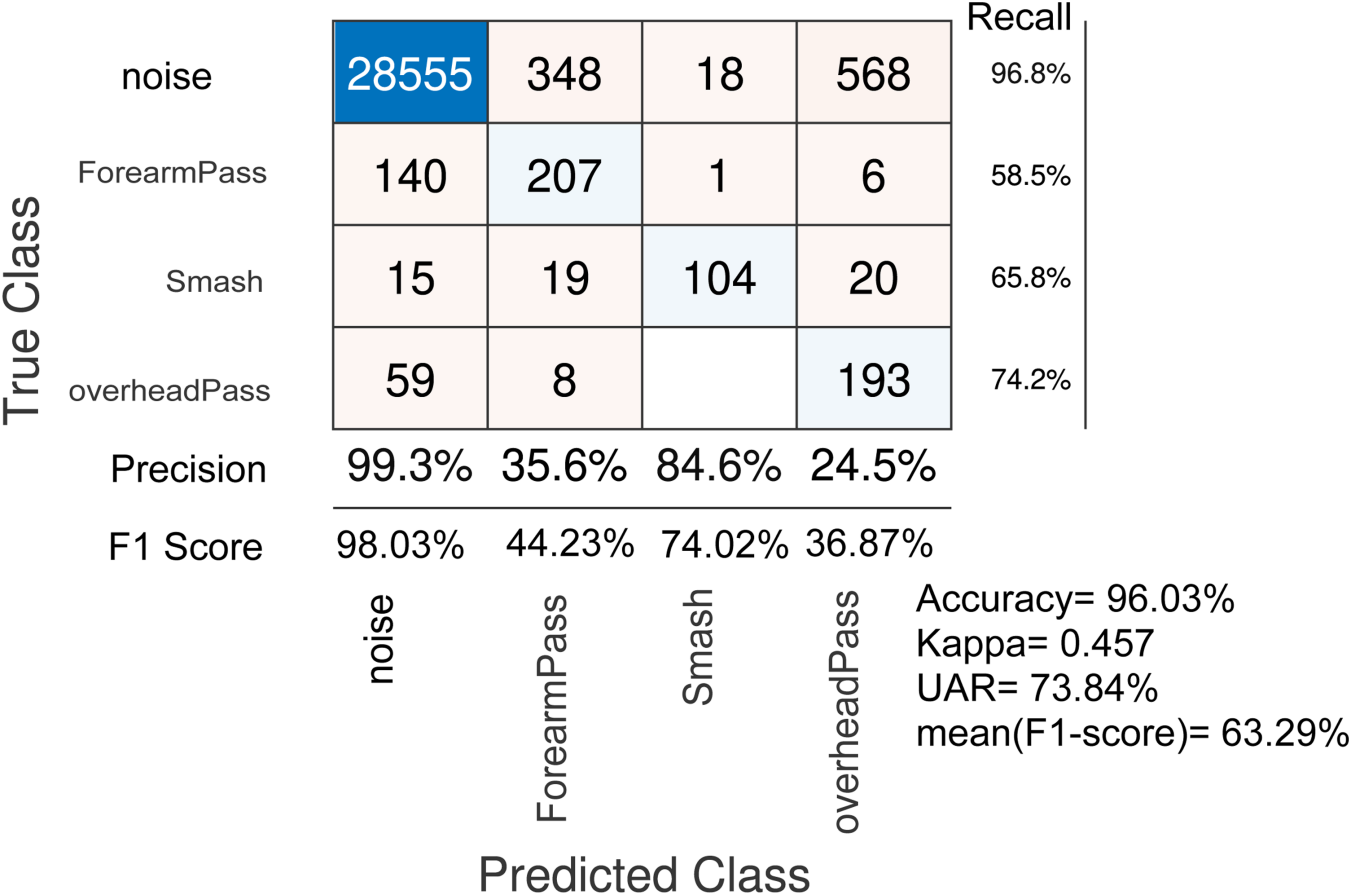
Action Recognition through ADR: leave one subject out cross-validation Results without impact detection method and no frames/windows discarding.

For LOSO cross-validation, The ADR method is able to predict the volleyball actions with an accuracy of 96.03%, well over the blind guess of 25% as shown in Figure 6. The proposed architecture also achieved an unweighted average recall (UAR) of 73.84% and an averaged $F_{1}\;\text{Score}$ of 63.29% with a Kappa of 0.457. It is noted that the noise is identified with the highest recall of 96.8%, precision of 99.3% and $F_{1}\;\text{Score}$ of 98.1%. The overhead pass and smash actions achieved a recall of 74.2% and 65.8% respectively. We obtained low precision rates for the overhead pass (24.5%) and forearm pass (35.6%), indicating that these actions are often misclassified as noise by the classifiers.

**Figure 7:**
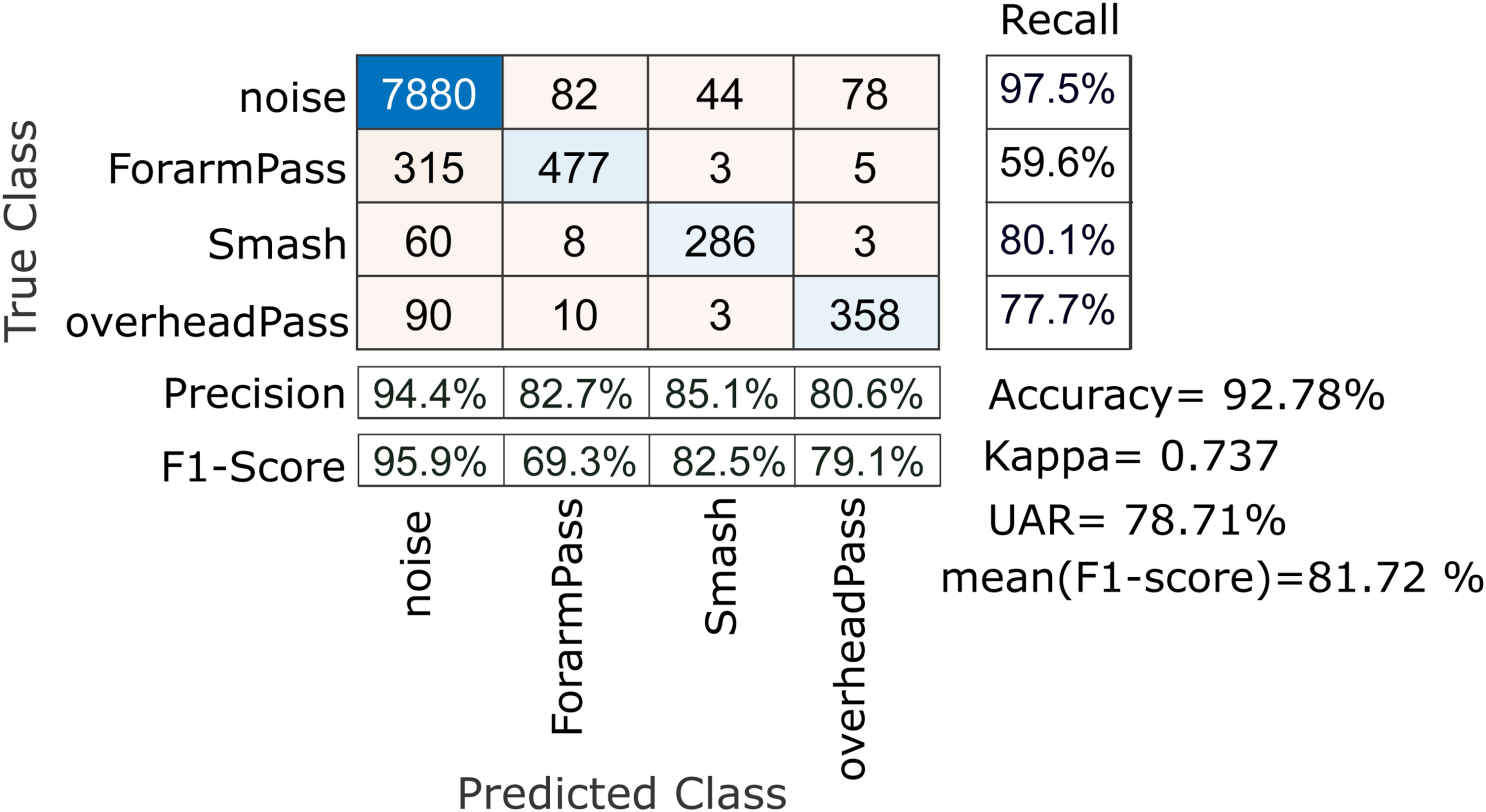
Action Recognition through CNN using raw signal: 10-fold cross-validation results with impact detection method.

In 10-Fold cross validation, CNN architecture is able to predict the volleyball actions with an accuracy of 92.78%, well over the blind guess of 25% and majority vote of 83.32%. The proposed architecture also achieved an unweighted average recall (UAR) of 78.71% and an averaged $F_{1}\;\text{Score}$ of 81.72% with a Kappa of 0.737. The UAR and averaged $F_{1}\;\text{Score}$ is the arithmetic mean of recall and $F_{1}\;\text{Score}$ of all four classes, respectively. The results of the cross-validation are shown in Figure 7. It is noted that the noise is clearly identified with the highest recall of 97.5%, precision of 94.4% and $F_{1}\;\text{Score}$ of 95.9%. The overhead pass and smash actions achieved a recall of 77.7% and 80.1% respectively. However, we also note that forearm pass has the lowest recall of 59.6%, indicating that the classification method is confusing the forearm with the noise class.

**Figure 8:**
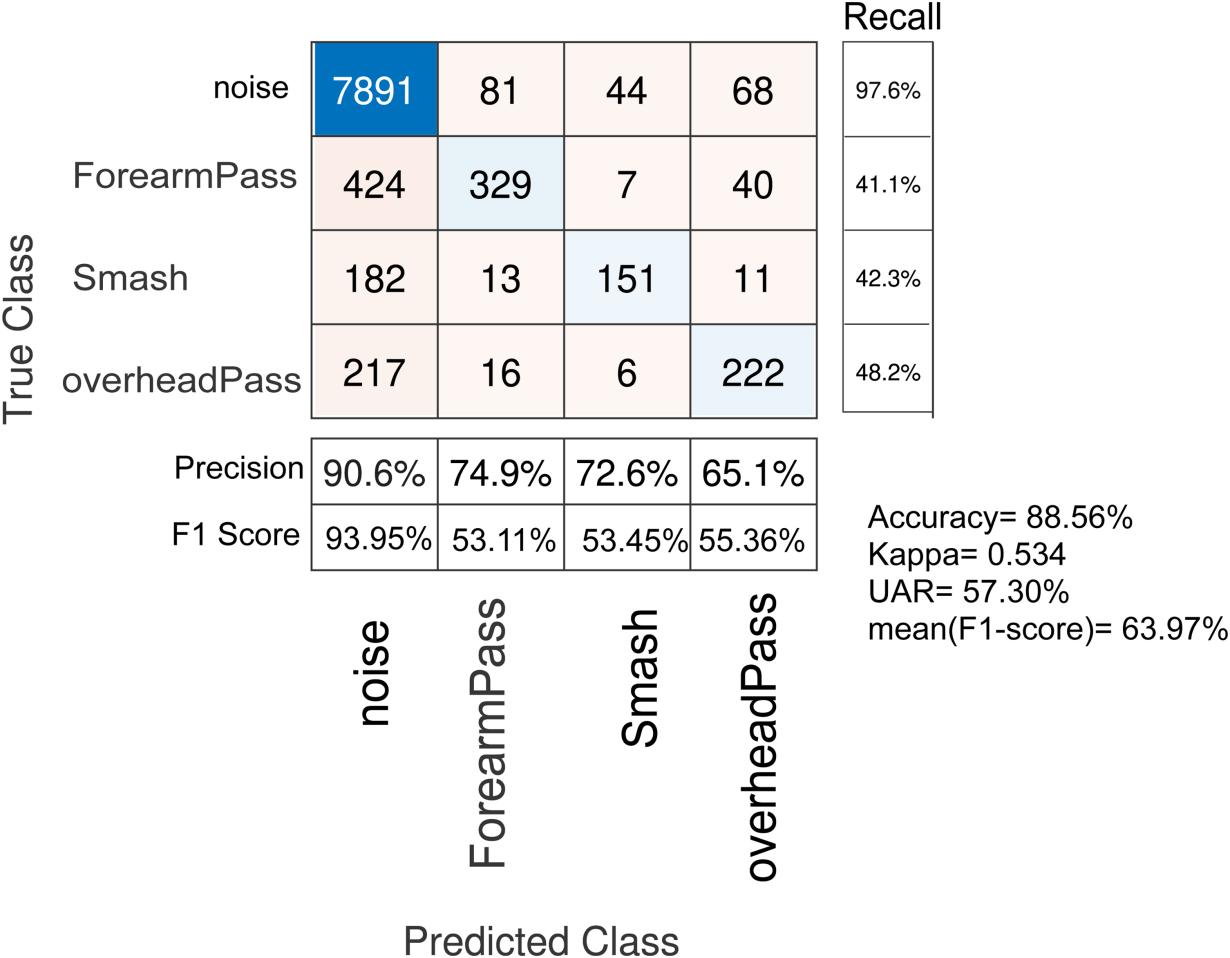
Action Recognition through CNN using raw signal: leave one out cross-validation results with impact detection method.

The performance of the CNN based classifier degraded when using LOSO cross-validation (see Figure 8) from a UAR of 78.71% to 57.30% indicating that the CNN and raw features are variant to subjective characteristics and more suitable for situations where a coach could enrol actions of a player to provide input for fine-tuning/retraining a CNN model.

The results from ADR and CNN indicate that CNN outperforms ADR in 10-fold cross-validation. The ADR and Statistical funcationls outperform CNN in LOSO cross-validation setting, indicating that statistical features and the ADR method are invariant to subjective characteristics.

The CNN approach incorporates an impact detection method to preprocess the data, potentially leading to overlapping frames during classification, especially considering that the number of frames is higher for CNN, except for noise frames. In contrast, the statistical functions and ADR methods process the data as a continuous one-second signal without overlap and without discarding any frames/windows, which reduces the risk of overfitting through dimensionality reduction.

These results show that it is possible to develop some level of automatic detection of relevant volleyball actions within the context of our real-time game platform. This suggests that this platform can be developed further in fruitful directions. However, clearly, the recognition results are not yet equally good enough for all types of interaction. The chance that a full sequence of Bump-Set-Spike will not be recognised because one of its constituent actions is not detected is too high to make the game work in its originally intended form: a full sequence has an a priori likelihood of only 67.87% Unweighted Average Recall (UAR), (3, 38) of being correctly identified. Therefore, until the recognition rates have been improved enough, it would probably be more effective to reward the occurrence of Set and Spike actions in isolation, gradually increasing the complexity and aesthetics of the visualisations depending on the rate of Set and Spike actions, rather than only making a significant change to the visualisations upon successful detection of the full triple set of actions.

In the next section, we will delve deeper into the implications of these results and discuss how the implementation of game scenarios should be precisely targeted to align with the expected performance of the individual real-time recognition modules.

## 7 Conclusion and future direction

We have described an end-to-end architecture for a modular system designed to be capable of both real-time (online) interactive training and data collection for sports training. The system can be configured to use different hardware e.g., IMUs from different manufacturers such as Xsens MTw Awinda (46) or x-io NG-IMUs (138) in combination with experimental pressure sensitive display floor.

The proposed architecture was implemented for a practical case study for interactive training of “Bump Set Spike” for volleyball players. The implemented system is used by other researchers to develop interactive games for sports training and physical education. Experimentation was also performed on volleyball data collected while players played volleyball during their regular training sessions. The trained deep-learning model results are highly encouraging, and show the applicability of the implemented system in real-time training scenarios. As with additional data collection, the model can be updated and due to the modular nature of the system, it allows easy update of the model or to even use different models by easily re-configuring the system.

Yet, performance is clearly not perfect. The efficacy of our detection and classification modules is contingent upon the precise application of their results. Even if our system’s recall or precision rates are not optimal, the “Events of Interest Exploration” system can still provide substantial assistance. This is because the retrieved training episodes, despite potential incompleteness or inclusion of irrelevant fragments, can yield valuable insights. However, in the case of interactive training like the Bump-Set-Spike module, frequent misfires of visual embellishments can undermine the system’s purpose.

In general, this implies that the classification components within the SSE platform need to be adjusted to prioritize false positives, false negatives, and the confusion of certain classes over others. The adjustment depends on the specific objectives of the application [see, for example, Beenhakker et al. (38)]. Specifically for the Bump-Set-Spike example, however, this also entails considering rewarding individual events rather than the entire bump-set-spike sequence. This approach helps mitigate cumulative errors in detecting the complete sequence. Therefore, the design of the application should be meticulously tailored to accommodate the performance characteristics of the available detection and recognition components.

The presented system is designed to serve as a platform not only for practical use by sports trainers but also for further interdisciplinary research. For instance, we plan to assess the system with coaches and players to gauge their interaction with it and explore ways to enhance the design for creating additional training games. Additionally, we aim to evaluate the system with various hardware configurations for interactive sports training. Lastly, we intend to conduct further experiments utilizing deep learning techniques to enhance the performance of the models for action classification.

## Data Availability

The raw data supporting the conclusions of this article will be made available by the authors, without undue reservation.

## Footnotes

^1^loosely based on https://datascience.berkeley.edu/about/what-is-data-science/ and the CRISP-DM process model for data mining by Wirth and Hipp (39).

^2^http://web3.bilkent.edu.tr/enterface19/—-last accessed March 2024
